# Genomic and Virulence Characterization of Intrauterine Pathogenic *Escherichia coli* With Multi-Drug Resistance Isolated From Cow Uteri With Metritis

**DOI:** 10.3389/fmicb.2018.03137

**Published:** 2018-12-17

**Authors:** Zhengxin Ma, Amber Ginn, Minyoung Kang, Klibs N. Galvão, Kwangcheol Casey Jeong

**Affiliations:** ^1^Emerging Pathogens Institute, University of Florida, Gainesville, FL, United States; ^2^Department of Animal Sciences, Institute of Food and Agricultural Sciences, University of Florida, Gainesville, FL, United States; ^3^Department of Large Animal Clinical Sciences, College of Veterinary Medicine, University of Florida, Gainesville, FL, United States; ^4^D. H. Barron Reproductive and Perinatal Biology Research Program, University of Florida, Gainesville, FL, United States

**Keywords:** intrauterine pathogens, *Escherichia coli*, dairy cows, extended spectrum β-lactamase, metritis

## Abstract

Metritis is a major disease in dairy cows causing animal death, decrease of birth rate, milk production, and economic loss. Antibiotic treatment is generally used to treat such disease but has a high failure rate of 23–35%. The reason for the treatment failure remains unclear, although antibiotic resistance is postulated as one of factors. Our study investigated the prevalence of extended spectrum β-lactamase (ESBL) producing bacteria in uterine samples of cows with metritis and characterized the isolated intrauterine pathogenic *Escherichia coli* (IUPEC) strains using whole genome sequencing. We found that the cows with metritis we examined had a high percentage of ESBL producing IUPEC with multi-drug resistance including ceftiofur which is commonly used for metritis treatment. The ESBL producing IUPEC strains harbored versatile antibiotic resistance genes conferring resistance against 29 antibiotic classes, suggesting that transmission of these bacteria to other animals and humans may lead to antibiotic treatment failure. Furthermore, these strains had strong adhesion and invasion activity, along with critical virulence factors, indicating that they may cause infectious diseases in not only the uterus, but also in other organs and hosts.

## Introduction

Metritis is one of the major global bacterial diseases of dairy cattle and is a prevalent reproductive disease characterized by an abnormally enlarged uterus, a watery red-brownish uterine discharge and fever within 21 days after parturition (Sheldon et al., 2006). It mainly affects dairy cows in the early postpartum period. Metritis decreases milk production, decreases conception rate, and increases culling and death (Drillich et al., 2001). Metritis affects approximately 20% of lactating dairy cattle with a range from 8% to more than 40% (Goshen and Shpigel, 2006; LeBlanc, 2008). The direct economic cost to the US dairy industry associated with this disease is approximately $600 million annually (Overton and Fetrow, 2008). The pathogens associated with metritis include *Escherichia coli*, *Trueperella pyogenes*, *Fusobacterium necrophorum*, *Fusobacterium nucleatum, Bacteroides pyogenes*, and *Porphyromonas levii* (Sheldon et al., 2009; Jeon et al., 2015; Cunha et al., 2018). The pathogens need essential pathogenicity traits including adhesion (Torres et al., 2005) and invasion (Sheldon et al., 2010) to host endometrial cells, motility mediated by flagella (Lane et al., 2007), and toxins like lipopolysaccharide (LPS) (Wolf, 1997) to manifest the disease. *E. coli* has been described as the main pathogen initiating postpartum uterine infection and disease (Bicalho et al., 2010; Sheldon et al., 2010).

Antibiotic treatment is used to treat animals with metritis, and the most common antibiotic is ceftiofur, a third generation cephalosporin that does not require milk withdrawal because the residue in milk is below the tolerance level for human consumption (Chenault et al., 2004). Ceftiofur is effective against both Gram-negative and Gram–positive pathogens (Collatz et al., 1990). However, approximately 25% of the cows fail to cure after being treated with ceftiofur (Chenault et al., 2004; McLaughlin et al., 2012). Although other antibiotics including ampicillin and tetracycline have been tested to treat metritis but they have not enhanced efficiency of curing metritis (Goshen and Shpigel, 2006; Lima et al., 2014). Multiple hypotheses have been proposed for the low cure rate of systematic administration of antibiotics, such as immunosuppression, periparturient disorders, and intrauterine placement of chemical compounds depressing the defense mechanism of cows (Smith et al., 1998). In addition, it was postulated that the continuous use of antibiotics in dairy farms attributed to the rise of antimicrobial resistance, especially to the commonly used antibiotic therapies, thereby leading to the ineffectiveness of antibiotics (Santos et al., 2010). However, the reason why a large proportion of animals do not respond to antibiotic treatment remains unclear.

Resistance to cephalosporins has been associated with bacteria carrying β-lactamases encoding genes, such as *bla*_TEM_, *bla*_SHV_, *bla*_CTX-M_, *bla*_CMY -2_, and *bla*_ampC_ (Zhao et al., 2001; Li et al., 2007). The plasmid-mediated CMY-2 type genes have been found in animal isolates extensively (Zhao et al., 2001; Donaldson et al., 2006; Chander et al., 2011). Isolates harboring TEM type β-lactamases usually have resistance to penicillin, but the resistance to expanded-spectrum cephalosporins was also reported (Poirel et al., 2004). In addition, CTX-M type extended spectrum β-lactamases (ESBLs) have been increasingly detected and confer resistance to expanded-spectrum cephalosporins, including cefotaxime, ceftazidime, and cefepime (Bonnet, 2004). In the UK, it was reported that in a dairy farm, 31/48 calves and 2/60 cows were positive for *E. coli* encoding the CTX-M gene in their fecal samples (Liebana et al., 2006). In Germany, one study found 86.7% of farms, including fecal and environmental samples, were positive for ESBL producing *E. coli*, most of which harbored CTX-M genes (Schmid et al., 2013). In addition, an extra-intestinal pathogenic *E. coli* (ExPEC) isolated from the uterus of a cow with metritis, MS499, had resistance to β-lactams, tetracyclines, and macrolides (Goldstone et al., 2014). Other pathogens resistant to β-lactam antibiotics, including *Listeria monocytogenes*, *Staphylococcus aureus*, and *Enterococcus* spp., have been also isolated from dairy cattle (Srinivasan et al., 2005; Tenhagen et al., 2006). Therefore, although there are multiple factors that may lead to treatment failure, we hypothesized that antimicrobial resistance is one of the key factors of this unfavored result. However, the prevalence of ESBL producing *E. coli* in the uterus of cows with metritis has not been reported. In this study, we focused our attention on the frequency of ESBL-producing *E. coli* in the uteri of cows with metritis and further characterized their antimicrobial resistance and virulence factors by whole genome sequencing and comparative genomics.

## Materials and Methods

### Animal Management

All animal procedures were approved by the University of Florida Institutional Animal Care and Use Committee (IACUC Protocol #: 201207405). Dairy Holstein cows were housed in freestall barns. The cows were fed twice daily with a mixed ration formulated to meet the nutrient requirements of a lactating cow weighing 650 Kg and producing 45 Kg of 3.5% fat-corrected milk per day. The animals were housed in the same farm, except one cow, the source of KCJ852.

### Uterine Sample Collection

Metritis was diagnosed by the presence of red-brownish fetid uterine discharge postpartum. Uterine swab samples were collected for individual animals (*n* = 24) after clinical diagnosis of metritis using the procedure previously described (Jeon et al., 2016). Briefly, after disinfection of the perineum area of cows with 70% ethanol, a sterile pipette containing a sterile cotton swab was introduced to the cranial vagina. To avoid vaginal contamination of the swab, the plastic sheath containing the pipette was directed into the cervix and then to the uterus. The plastic pipette was then protruded through the plastic sheath, then the sterile swab was exposed and rolled against the uterine wall to collect sample. The swab was pulled inside the pipette then the pipette pulled inside the plastic sheath and removed from the cow. Swabs were transferred to a 15 mL tube on ice and delivered to the laboratory within 4 h. The samples were collected from the cows ranging from day 2–7 after parturition upon diagnosis of metritis.

### Identification of Cefotaxime Resistant Pathogens

The swab samples were enriched in tryptic soy broth overnight in the presence of cefotaxime to select ESBL positive bacteria. Next, the samples were plated on MacConkey agar with cefotaxime (4 mg/L) and incubated at 37°C to select cefotaxime resistant colonies. Five colonies from each uterine sample grown were purified on the same media and confirmed if they were *E. coli* by striking them on ChromAgar *E. coli* (CHROMagar, France). The presence of ESBL genes (*bla*_TEM_, *bla*_CTX-M_, *bla*_CMY_) was tested by PCR using primers previously described (Mir et al., 2016). Fifteen isolates purified from 3 swab samples carried *bla*_CTX-M_ gene and one isolate without ESBL genes were confirmed as *E. coli*; these isolates were then further characterized. One ESBL negative sample was plated on ChromAgar *E. coli* directly to isolate a non-ESBL endometrial pathogenic *E. coli*.

### Adherence and Invasion Assay

Four cell lines were used in this study. Caco-2, HEK293T and Hep2 cells were cultured in Dulbecco’s modified Eagle medium (DMEM, Corning Incorporated, Corning, NY, United States) supplemented with 10% fetal bovine serum (FBS, Hyclone, Logan, UT, United States). Bovine endometrial epithelial cells (Sigma-Aldrich, St. Louis, MO, United States) were cultured in Bovine Endometrial Growth Media (Sigma-Aldrich, St. Louis, MO, United States). The cells were cultured to confluency. Then approximately 10^5^ cells were seeded into 24-well plates and incubated overnight at 37°C and 5% CO_2_. The adherence assay was conducted following previously described protocol with modifications (Amigo et al., 2015). Gentamicin protection assay was used for invasion test (Falkow et al., 1987). Bacteria (KCJ4393 [DH5α], KCJ696 [the *eae* deletion mutant of *E. coli* EDL933], and KCJ698 [the *tir* deletion mutant of *E. coli* EDL933] as negative controls, *E. coli* EDL933 as a positive control, KCJ852, KCJ3819, KCJ3823, and KCJ3859) were cultured overnight at 37°C in Luria Bertani (LB) broth and washed with sterile phosphate buffered saline (PBS) three times. The final bacterial pellet was diluted to 10^6^ CFU/mL and resuspended in cell culture media and added to each well. For adherence assay, the mixture of cell and bacterial culture was incubated at 37°C and 5% CO_2_ for 3 h. After the first incubation, the media was replaced by DMEM and the cell culture was incubated for another 3 h. Then the media was removed and cells were washed three times with PBS, and lysed by 1 mL of 0.1% Triton X-100 in PBS. The culture was serially diluted and plated on LB agar plates to enumerate the bacteria. For the invasion assay, the cultures were incubated at 37°C and 5% CO_2_ for 4 h. Next, the supernatant was removed and the cells were washed with PBS three times. Media containing 50 μg/mL gentamicin was added to each well and the culture was incubated for another 1.5 h. After incubation, the cells were washed and the bacteria were enumerated as described in adherence assay.

### Minimal Inhibitory Concentration Test and Antimicrobial Susceptibility Test

MIC test of cefotaxime was conducted with the 15 strains carrying *bla*_CTX-M_ genes and one ESBL negative strain (KCJ852). MIC was determined using broth microdilution method according to CLSI guidelines (CLSI, 2018). KCJ1409 (Mir et al., 2016), an ESBL-producing *E. coli* isolated from human was included as a positive control and DH5α, a non-pathogenic *E. coli* was included as a negative control.

Antimicrobial susceptibility test (AST) was further performed on each representative strain (KCJ3819, KCJ3823, and KCJ3859) from CTX-M positive samples (*n* = 3) and one ESBL negative strain (KCJ852). The standard Kirby Bauer disk diffusion method on Mueller Hinton agar was used. The control strains used for the AST were *E. coli* (ATCC 35401), *S. aureus* (ATCC 25923) and *Pseudomonas aeruginosa* (ATCC 27853). The antimicrobial disks used are listed below: Amikacin (K; 30 μg), Ampicillin (A; 10 μg), Amoxycillin/Clavulanic acid (X; 30 μg), Sulfisoxazole (Z; 0.25 mg), Ceftiofur (R; 30 μg), Chloramphenicol (C; 30 μg), Cephalothin (F; 30 μg), Gentamicin (G; 10 μg), Nalidixic acid (N; 30 μg), Streptomycin (S; 10 μg), Sulfamethoxazole/trimethoprim (M; 23.75 μg/1.25 μg), and Tetracycline (T; 30 μg) (BD, United States).

### Whole Genome Sequencing and Phylogenetic Tree Analysis

Representative strains (*n* = 13) from CTX-M positive samples and the non-ESBL strain KCJ852 were sequenced by Illumina MiSeq. Pure genomic DNA was extracted from each isolate using the DNeasy blood and tissue kit (Qiagen, Valencia, CA, United States). High sensitivity double stranded DNA quantification was performed using the Qubit^®^ fluorometer assay and the DNA was diluted to 0.2 ng/μl and used as input DNA for tagmentation with Illumina’s Nextera^®^XT DNA library sample preparation kit. Sequencing was performed using an Illumina MiSeq with cartridges providing 2 × 250 paired-end read coverage. *De novo* assemblies were performed using SPAdes (Bankevich et al., 2012) and fastq files were trimmed for quality and length using Sickle (Joshi and Fass, 2011) prior to assembly. Alignment of the *de novo* assembles was performed using progressiveMauve 2.4.0 (Darling et al., 2010). Single nucleotide polymorphisms (SNPs) were extracted from the whole genome alignment using MEGA 6.0 (Tamura et al., 2013) and a maximum likelihood tree was constructed using 1000 bootstrap iterations and automatic model selection with IQ-TREE (Trifinopoulos et al., 2016). To determine the best fitting roots for maximum likelihood phylogenies, TempEst was used and final tree annotations were made using FigTree (Rambaut et al., 2016). Whole genome alignments among strains in the same cluster were performed using progressiveMauve. Prophages were identified by PHAST (Zhou et al., 2011).

#### Virulence and Antibiotic Resistance Gene Identification

Genomes were annotated using the Virulence Factor Database (VFDB) to identify virulence and antibiotic resistance genes (Chen et al., 2005) through PATRIC (Wattam et al., 2014). The Center for Genomic Epidemiology (CGE) database was used to identify the multi-locus sequence type (MLST), serotype, and plasmid replicons for each isolate (Larsen et al., 2012; Carattoli et al., 2014; Joensen et al., 2015). The Comprehensive Antibiotic Resistance Database (CARD) was also employed for discovery of additional antibiotic resistance genes (McArthur et al., 2013).

### Statistical Analysis

All experiments were carried out in triplicate. For MIC, the data were reported as mean ± SEM. For adherence and invasion assays, data were analyzed using the GLM procedure of SAS version 9.1 (SAS Institute Inc., Cary, NC, United States). Average values with standard error of means were reported. *P* ≤ 0.05 were considered as statistically significant.

## Results

### High Frequency of Cephalosporin Resistance in Cows With Metritis in the Farm Sampled

Dairy cows with metritis are usually treated with ceftiofur but the failure rate of such treatment is 23–35% (Chenault et al., 2004). To investigate if cephalosporin resistance is one of the main reasons affecting the treatment failure rate, we first evaluated the prevalence of cephalosporin resistance by plating uterine swab samples from cows with metritis on media containing cefotaxime, which is a third generation cephalosporin used to select ESBLs. Out of 24 uterine swab samples tested, 17 samples had colonies growing on media in the presence of cefotaxime, thus the percentage of cefotaxime resistance was 70.8%. PCR was conducted using total microbiome DNA to identify if the microbiota in these samples carry extended-spectrum β-lactamase (ESBL) genes. We found that CMY-2 β-lactamase gene (*bla*_CMY_) was detected in 9 samples, while ESBLs (*bla*_CTX_) was detected in 3 samples. One samples carried both *bla*_CMY_ and *bla*_TEM_, and another sample carried a combination of *bla*_CMY_ and *bla*_CTX_. By purifying colonies on ChromAgar *E. coli*, all the colonies presented blue color, suggesting all the ESBL-positive isolates were *E. coli*.

As CTX-M family have become the most common type of ESBLs with high clinical importance, we further purified 5 *E. coli* isolates from each CTX-M-positive samples and one ESBL-negative sample for comparison purpose from the cow uterus (Table 1). KCJ852 was from an ESBL negative cow, while KCJ3815 - 3819, KCJ3820 - 3824, and KCJ3855 - 3859 were from ESBL-positive cows, respectively.

**Table 1:** Information of strains isolated from uterine samples.

Strain name	Source	Animal	Disease	Lactation
KCJ852	Uterine mucus	Cow 1	Metritis	Not available
KCJ3815	Uterine mucus	Cow 2	Metritis	3
KCJ3816	Uterine mucus	Cow 2	Metritis	3
KCJ3817	Uterine mucus	Cow 2	Metritis	3
KCJ3818	Uterine mucus	Cow 2	Metritis	3
KCJ3819	Uterine mucus	Cow 2	Metritis	3
KCJ3820	Uterine mucus	Cow 3	Metritis	2
KCJ3821	Uterine mucus	Cow 3	Metritis	2
KCJ3822	Uterine mucus	Cow 3	Metritis	2
KCJ3823	Uterine mucus	Cow 3	Metritis	2
KCJ3824	Uterine mucus	Cow 3	Metritis	2
KCJ3855	Uterine mucus	Cow 4	Metritis	5
KCJ3856	Uterine mucus	Cow 4	Metritis	5
KCJ3857	Uterine mucus	Cow 4	Metritis	5
KCJ3858	Uterine mucus	Cow 4	Metritis	5
KCJ3859	Uterine mucus	Cow 4	Metritis	5


### Intrauterine Pathogenic *E. coli* Had Distinct Clonal Groups

**FIGURE 1:**
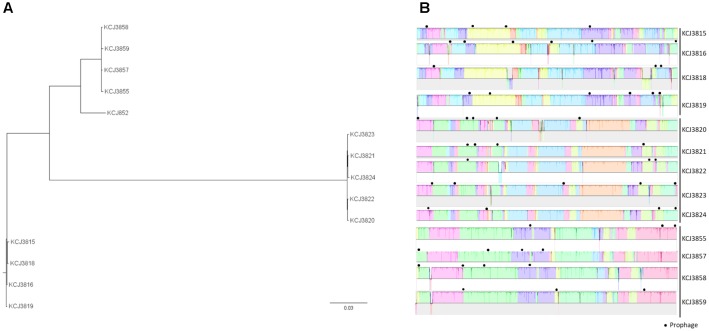
Comparison of intrauterine pathogenic *E. coli* from different cows. **(A)** Phylogeny of intrauterine pathogenic *E. coli* isolated from this study. **(B)** Genome alignment of CTX-M positive intrauterine pathogenic *E. coli*. The chromosomal sequences of IUPECs collected from one animal were aligned by progressiveMauve. The background color gradient indicates homologous regions across strains. Prophages predicted by PHAST were labeled by black solid circles.

To evaluate the genomic relationship among the intrauterine pathogenic *E. coli* (IUPEC) isolates from uterine samples, phylogenic analysis was conducted after whole genomic sequencing. The genome size of 13 strains varied from 4.7 to 5.0 Mb, with KCJ852 being the shortest and KCJ3820-3825 being the longest. It was observed that the isolates collected from each of the cow uterine samples were clustered into the same clade (Figure 1A), suggesting that there was no transmission of isolates between animals although they were housed together. The isolates from cow #3 (KCJ3820-3824) showed longer phylogenetic distance to other isolates, demonstrating substantial genomic diversity from the other isolates. Interestingly, the ESBL-negative strain, KCJ852 (Ginn et al., 2016), was clustered closely to one clade of CTX-M-positive isolates, even though they were not collected from the same farm. To support the findings in Figure 1A, isolates in each clade were aligned by progressiveMauve (Figure 1B) to compare genome rearrangement and architecture. Within each clade, the chromosomal DNA architectures of the assembled isolates were similar to each other, suggesting they were clonal variants. However, variation in the number and content of the prophages acquired by each isolate was present. None of the isolates had the same set of prophages.

### Multi-Drug Resistance of the Uterine Isolates

**FIGURE 2:**
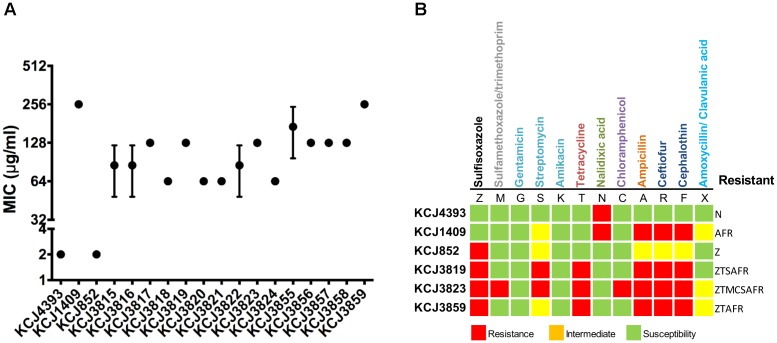
The antimicrobial resistant profile of intrauterine pathogenic *E. coli* from this study and reference human clinical isolates. **(A)** Minimal inhibitory concentration test. Bars represent the mean ± SEM of three experiments. **(B)** Antimicrobial susceptibility test. The antibiotics in different colors indicate they belong to different antibiotic classes. Red squares indicate the strain was resistant to the antimicrobial; yellow squares indicate the strain was intermedium resistant to the antimicrobial; green squares indicate the strain was susceptible to the antimicrobial. KCJ4393: *E. coli* DH5α.

**FIGURE 3:**
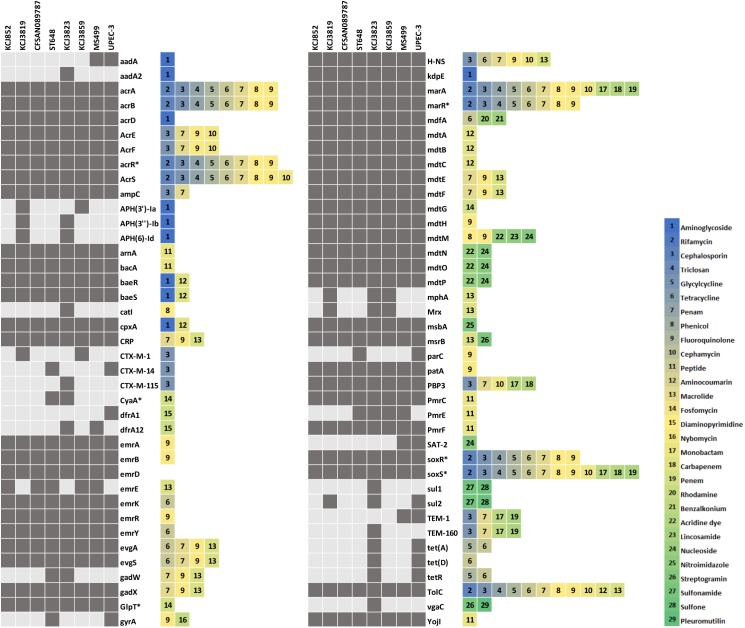
The antibiotic resistance gene profile of intrauterine pathogenic *E. coli* from this study and reference human clinical isolates. Dark grey boxes indicate that the gene of the isolate has > 70% similarity to the gene in the Comprehensive Antibiotic Resistance Database.

In order to address the question of if these IUPEC isolates were associated with treatment failure, we assessed multidrug resistance by MIC and AST. For MIC of cefotaxime (Figure 2A), if the level is equal or above 64 μg/mL, the strain is considered clinically relevant in human medicine. According to CLSI, if the MIC is above 4 μg/mL, the isolate is resistant to cefotaxime. The CTX-M negative isolates, *E. coli* DH5α and KCJ852, did not show resistance (MIC = 2 μg/mL), whereas the MIC against the CTX-M gene carriers, including the positive control KCJ1409, were higher than 64 μg/mL. The highest resistance was observed for the strains isolated from cow #4 (KCJ3855 – 3859), as the MIC ranging from 128 to 256 μg/mL, confirming the CTX-M positive isolates were highly resistant to cefotaxime. The AST of 12 different types of antibiotics, belonging to 9 classes, against representative strains were summarized in Figure 2B. The non-pathogenic *E. coli* DH5α had resistance to only one antibiotic, nalidixic acid, which inhibits a subunit of DNA gyrase and topoisomerase IV. The CTX-M negative strain KCJ852 showed resistance to one antibiotic, sulfisoxazole. The positive control KCJ1409 showed resistance to four antibiotics. All the IUPECs carrying CTX-M had resistance to at least five antimicrobials across multiple drug classes. KCJ3823 had resistance against eight antibiotics. The CTX-M positive strains were highly resistant to all the β-lactam antibiotics tested (ampicillin, ceftiofur, and cephalothin), whereas none of them were resistant to gentamicin, amikacin, or nalidixic acid. All the IUPEC strains showed multidrug resistance, shown by the AST results (Figure 2B). Notably, all of these ESBL-producing *E. coli* harbored resistance against ceftiofur, the antibiotics commonly used for the treatment of metritis. Taken together, these data indicate that it is possible that these cefotaxime resistant IUPEC isolates are associated with treatment failure of metritis using ceftiofur. Therefore, our data indicate that strains isolated from cows with uterine diseases confer multidrug resistance against 5–8 different antibiotics while carrying potential drug resistance genes which may provide resistance against a wide range of antibiotics. Furthermore, 78 antibiotic resistance genes (ARGs) responsible for resistance of 29 antibiotic classes were identified through the Comprehensive Antibiotic Resistance Database (CARD) (McArthur et al., 2013), while 71 genes were identified in the IUPEC strains (Figure 3). The majority of the resistance genes had functions related to efflux pump (*acrABDEFRS*, *emrABDEKRY*, *evgAS*, *gadWX*, *marAR*, *mdtABCEFGHMNOP, msbA, msrB, soxRS, tetADR, and yojI*) followed by antibiotic inactivation enzyme such as β-lactam resistance genes (*ampC*, CTX-M, TEM). KCJ3819 and 3859 had CTX-M-1 while KCJ3823 had CTX-M-115. All the IUPEC strains isolated had *amp*C. KCJ852 harbored less ARGs compared with other strains. In general, our IUPEC strains had very similar ARG profiles compared with the clinically important *E. coli* strains.

### Genomic Characterization of the Uterine Isolates

To evaluate the importance of these IUPEC strains, the genomic characteristics were compared with other representative clinical pathogenic *E. coli* isolates (ST648, UPEC-3, and CFSAN029787) and another IUPEC strain (MS499). Genome information of these strains were obtained from NCBI. These strains are listed in Table 2. *E. coli* ST648, serotype O51:H4, was isolated from pleural effusion of a patient with empyema thoracis in Beijing, China. UPEC-3, an *E. coli* O9:H9, was isolated from a patient with an urinary tract infection in Washington, DC, United States. CFSAN029787 was an enteroinvasive *E. coli* belonging to serotype O96:H19 isolated from stool samples in Milan, Italy (Table 3). The IUPECs from the same cow had the same serotype and multilocus sequence type (MLST), and the same set of plasmids (Table 3), but isolates from different samples had different serotype and MLST profiles. KCJ852 had the same serotype as KCJ3855 – 3859, but not MLST type. KCJ852 had only one plasmid replicon, IncII, whereas the other IUPEC strains shared two common plasmid replicons, IncFII and IncFIB. Based on the analysis using Center for Genomic Epidemiology (CGE) (Larsen et al., 2012; Carattoli et al., 2014; Joensen et al., 2015), KCJ3815 – 3819 and 3855 – 3859 had CTX-M-1, whereas KCJ3820 – 3824 had CTX-M-124 and TEM-1B. None of these IUPEC isolates showed a similar genomic profile compared with the reference strains. In all respects, the unique profile of isolates from each sample indicated no transmission of IUPEC strains among the cows had occurred; however, the possibility exists that the plasmids were transmitted among the strains.

**Table 2:** Strains used in this study.

Strain name	NCBI accession	Description of isolation source^b^	Reference
DH5α	N/A^a^		NEB^@^5-alpha competent *E. coli* (High Efficiency)
KCJ1409	SAMN05789710		Mir et al., 2016
ST648	SAMN02800875	ExPEC	Genbank
EDL933	SAMN02604092	EHEC	Genbank
MS499	SAMN02839404	IUPEC	Genbank
UPEC-3	SAMN02802119	UPEC	Genbank
CFSAN029787	SAMN03612246	EIEC	Genbank
KCJ852	SAMN04396104	IUPEC	This Study
KCJ3815	SAMN04396076	IUPEC	This Study
KCJ3816	SAMN04396077	IUPEC	This Study
KCJ3817	N/A	IUPEC	This Study
KCJ3818	SAMN04396078	IUPEC	This Study
KCJ3819	SAMN04396079	IUPEC	This Study
KCJ3820	SAMN04396080	IUPEC	This Study
KCJ3821	SAMN04396081	IUPEC	This Study
KCJ3822	SAMN04396082	IUPEC	This Study
KCJ3823	SAMN04396083	IUPEC	This Study
KCJ3824	SAMN04396084	IUPEC	This Study
KCJ3855	SAMN04396085	IUPEC	This Study
KCJ3856	N/A	IUPEC	This Study
KCJ3857	SAMN04396086	IUPEC	This Study
KCJ3858	SAMN04396087	IUPEC	This Study
KCJ3859	SAMN04396088	IUPEC	This Study


*^a^N/A, not available. ^b^ExPEC, extraintestinal pathogenic E. coli; EHEC, enterohemorrhagic E. coli; IUPEC, intrauterine pathogenic E. coli; UPEC, uropathogenic E. coli; EIEC, enteroinvasive E. coli.*

**Table 3:** Characterization of intrauterine pathogenic *E. coli* isolates.

Strain name	Serotype	MLST^a^	Plasmid replicons	Beta-lactam resistance genes
ST648	O51:H4	648	IncFIA, IncFIB, IncFII, Col	TEM-1B
MS499	H16:O23	453	IncFII, IncFIB	TEM-1C
UPEC-3	O9:H9	162	IncFIB, IncFIC, IncB/O/K/Z	CTX-M-14, TEM-1B
CFSAN029787	O96:H19	99	IncFII, IncFIB, IncQ1, IncFII	-
KCJ852	O8:H19	708	Incl1	-
KCJ3815	O54:H2	2328	IncFII, IncFIB, IncN, ColRNAI	CTX-M-1
KCJ3816	O54:H2	2328	IncFII, IncFIB, IncN, ColRNAI	CTX-M-1
KCJ3818	O54:H2	2328	IncFII, IncFIB, IncN, ColRNAI	CTX-M-1
KCJ3819	O54:H2	2328	IncFII, IncFIB, IncN, ColRNAI	CTX-M-1
KCJ3820	:H42	648	IncFIA, IncFIB, IncFII, IncQ1	CTX-M-124, TEM-1B
KCJ3821	:H42	648	IncFIA, IncFIB, IncFII, IncQ1	CTX-M-124, TEM-1B
KCJ3822	:H42	648	IncFIA, IncFIB, IncFII, IncQ1	CTX-M-124, TEM-1B
KCJ3823	:H42	648	IncFIA, IncFIB, IncFII, IncQ1	CTX-M-124, TEM-1B
KCJ3824	:H42	648	IncFIA, IncFIB, IncFII, IncQ1	CTX-M-124, TEM-1B
KCJ3855	O8:H19	162	IncFIB, IncFII, IncN	CTX-M-1
KCJ3857	O8:H19	162	IncFIB, IncFII, IncN	CTX-M-1
KCJ3858	O8:H19	162	IncFIB, IncFII, IncN	CTX-M-1
KCJ3859	O8:H19	162	IncFIB, IncFII, IncN	CTX-M-1


*^a^MLST, multilocus sequence typing.*

**FIGURE 4:**
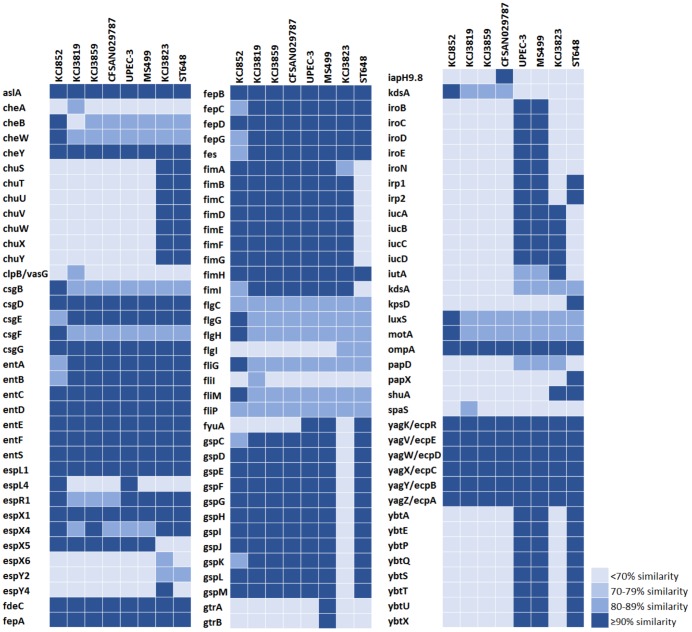
Virulence factor profile of intrauterine pathogenic *E. coli* from this study and reference human clinical isolates.

The virulence factors were identified for the same set of strains by Virulence Factor Database (VFDB) (Chen et al., 2005). The number of virulence genes IUPEC strains harbored varied from 65 to 70 (Figure 4). The IUPEC strains harbored important virulence factors including iron uptake (*ent*, *ybt*, and *fep* family), adherence *(fim*, *fli, yag/ecp*, and *flg* family), and invasion (*asl*A and *ompA)*. The most invasive strain in bovine endometrial cell line, KCJ3819 (see later section of the results), harbored unique genes *cheA*, *clpB/vasG*, *fliI*, and *spaS*. Particularly, *clpB/vasG*, *fliI*, and *spaS which* are related to Type 6 Secretion System (Shrivastava and Mande, 2008), flagella (Dreyfus et al., 1993), and Type 3 Secretion System (Zarivach et al., 2008), respectively. Again, the IUPEC strains showed similar virulence factor profiles compared with the clinical isolates, suggesting they could be transmitted to humans and cause human infectious disease.

### Intrauterine Pathogenic *E. coli* Had High Adhesive and Invasion Ability

**FIGURE 5:**
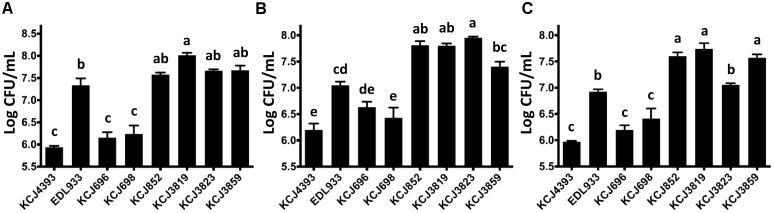
Adherence of intrauterine pathogenic *E. coli* to different cell lines. **(A)** Caco-2, **(B)** HEK293, **(C)** HEP2 cells. KCJ4393: *E. coli* DH5α; KCJ696: *eae* deletion mutant of EDL933; KCJ698: *tir* deletion mutant of EDL933; Bars represent the mean ± SEM of three experiments. Means with different letters differ (*P* < 0.05).

As adhesion is the first step for bacteria to colonize and cause pathogenicity, we tested the adherence ability of IUPEC strains in three human cell lines (Figure 5). The commensal *E. coli* strain, DH5α (KCJ4393) is known to have low adherence ability compared to pathogenic strains, thus it was included as a negative control. Oppositely, *E. coli* EDL933 (positive control) was reported to have high adherence activity. *eae* and *tir* are attaching and effacing virulence genes of *E. coli* O157:H7 EDL933 and play a key role in the adhesion to the host. Therefore, we included the deletion mutants of *eae* (KCJ696) and *tir* (KCJ698) as negative controls. In Caco-2 cells, the IUPEC strains, regardless if they carried ESBLs, adhered to the cells as efficiently as EDL933 (Figure 5A), indicating the strains were able to colonize in the intestinal tract. HEK293T is a human embryonic kidney cell line and HEP2 is a human cervix carcinoma cell line. The IUPEC strains were able to adhere to these two types of cells equal to or even more significantly than EDL933 (Figures 5B,C), showing the possibility of these strains to cause infection in different sites once invaded to the host.

**FIGURE 6:**
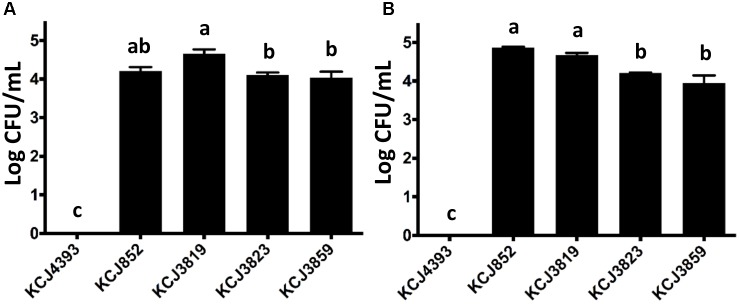
Invasion of intrauterine pathogenic *E. coli* to different cell lines. **(A)** Bovine endometrial epithelial and **(B)** Caco-2 cells. KCJ4393: *E. coli* DH5α; Bars represent the mean ± SEM of three experiments. Means with different letters differ (*P* < 0.05).

As the pathogenic *E. coli* associated with pelvic inflammatory disease have the ability to invade the endometrial epithelial cells, we conducted an invasion assay using a bovine endometrial epithelial cell line with representative isolates from each CTX-M-positive animal sample to confirm that the cephalosporin resistant isolates were IUPEC (Figure 6A). Also, a negative control, the non-pathogenic *E. coli* DH5α (KCJ4393), and KCJ852 were included. It was observed that *E. coli* DH5α did not have any invasive ability, while for the other four strains, significant numbers of bacteria invaded into the cells (>4 log CFU/mL). Among the pathogenic strains, KCJ3819 showed the greatest ability of invasion. These results indicate that these strains isolated from the uterus were etiological agents of metritis. Another invasion assay was further conducted with Caco-2 cells, which is a human intestinal epithelial cell line (Figure 6B). Similar results were observed that all the IUPEC strains showed high invasion abilities to the cells, suggesting that these strains may also cause infectious disease in the gastrointestinal tract.

## Discussion

It has frequently been reported that farm animals are reservoirs for ESBL producing *Enterobacteriaceae*. The prevalence of CTX-M-carrying *E. coli* on a dairy commercial farm using cephalosporin was reported as 39% (Dolejska et al., 2011). Also, dairy herds with ceftiofur use had an approximately 25 times higher chance to have ESBL producing *E. coli* compared with the herds that did not use ceftiofur (Tragesser, 2003). The prevalence of ESBL producing *E. coli* in the uterus of cows with metritis has not been reported. In our study, 58% of animals had ESBL producing *E. coli* in their uterine samples. This high percentage is likely to be associated with the continuous use of ceftiofur in the herd. A limitation of this study was that mainly one dairy farm was recruited. Therefore, the result may not represent the prevalence of ESBL producing *E. coli* in dairy farms overall. Further studies will be needed to compare the prevalence of ESBL carriers among the cattle population in different geographical regions. Moreover, it would be desirable to correlate the prevalence with a history of antibiotic use on individual farms.

The IUPEC isolates from each cow belonged to different MLST types. Isolates from cow #3 (KCJ3820 – KCJ3824) belong to sequencing type (ST) 648 (Table 3). Pathogenic *E. coli* ST648 strains have been isolated from human, companion animals and farm animals, and some of them carried ESBL genes including CTX-M-1, -3, -14, -15, and -61 (Peirano et al., 2012; Ewers et al., 2014). They have caused urinary and respiratory tract infections in human hosts (Ewers et al., 2014). They shared common virulence genes like other ExPECs harboring genes, including *papC*, *iutA*, *kpsMTII*, *sfa/foc*, and *afa/dra* (Johnson et al., 2003). Isolates from the uterus of cow #4 belonged to ST162. This ST type *E. coli* have also been found in humans, companion animals and livestock. ST162 has been frequently associated with urinary tract infections (Dierikx et al., 2012; Yuan et al., 2012). They were reported to carry ESBL type CTX-M-1 and TEM-1 (Dierikx et al., 2012). However, no information about ST708 and ST2328, which IUPEC isolates from cow #1 (KCJ852) and #2 (KCJ3815 -KCJ3819) belonged to respectively, can be found in the literature. In addition, the IUPEC isolates from different cows belong to different serotypes and MLST types, suggesting that there was low pathogen transmission among animals although the strains were isolated from the same farm at the same time. These data are consistent with the previous findings that IUPEC isolates did not cluster significantly by farms by random amplified polymorphic DNA-PCR cluster analysis (Bicalho et al., 2010). Sheldon et al. (2010) also reported that IUPEC strains were from various clusters by MLST analysis. This indicates that the strains gained antibiotic resistance either during parallel evolution or by plasmid acquisition. However, further studies with more sample numbers are needed to understand this phenomenon.

Interestingly, however, these isolates harbored two identical plasmid groups, IncFIB and FII (Table 3). IncFIB plasmid was frequently observed in avian pathogenic *E. coli* (Johnson et al., 2007) and IncFII plasmid was usually associated with *bla*_CTX-M_ gene (Hopkins et al., 2006). IncN plasmid was carried by isolates from both cow #2 and #4, which harbored CTX-M-1. IncN plasmid was reported to carry *bla*_CTX-M-1_ and the *E. coli* strains which carried IncN plasmid were isolated from a dairy farm (Dolejska et al., 2011). Thus, the resistance genes may disseminate among animals via bacterial conjugation system (de Been et al., 2014). The IUPEC strains in this study carried CTX-M-1 (KCJ3815-3819 and KCJ3855-3859) and CTX-M-115 (KCJ3820-3824, Figure 3). CTX-M-1 type β-lactamase was frequently observed in both food producing animal isolates and human clinical isolates (Eckert et al., 2004; Meunier et al., 2006; Cormier et al., 2016). CTX-M-115 type was detected less frequently, but it was also found in both clinical isolates (Munoz-Price et al., 2013; Pfeifer et al., 2016) and farm animals (Cormier et al., 2016). These data further support the possible transmission of the ARGs among animals and humans, especially through plasmid acquisition.

The IUPEC strains isolated in this study harbored essential virulence genes including adherence. For example, the fimbriae are encoded by the *fim* gene cluster, which are necessary for adhesion to host cell surfaces (Hultgren et al., 1991). The presence of *fimH* positive *E. coli* in uteri increases the chance of establishing and persistence of uterine infection by other pathogenic Gram-negative bacteria and reduces the reproductive performance (Bicalho et al., 2012). In addition, *E. coli* carrying type 1 fimbrial *fimH* detected at 1-3 days in milk was reported to be significantly associated with development of metritis and endometritis (Bicalho et al., 2012). In this study, all the IUPEC strains isolated were *fimH* positive, showing that they increase the risk of uterine disease. Although some virulence factors such as siderophores (*iroBCDE*/*iucABCD*) and iron uptakes (chu*STUVWXY*) were missing compared to the previously reported IUPEC strain, MS499 (Goldstone et al., 2014), all the IUPEC strains harbored adherence genes (Figure 4) and were able to adhere and invade into epithelial cells (Figures 5, 6) which is consistent with findings of other studies (Sheldon et al., 2010; Poole et al., 2017).

## Conclusion

The present study showed the high frequency of ESBL producing *E. coli* in the uteri of cows having metritis we sampled. The IUPEC isolates were adhesive and invasive to different cell lines; this provided a clear demonstration that they can cause disease at different sites of the host. These isolates had resistance to the common antibiotic treatment of metritis (ceftiofur) and, therefore, the high treatment failure of metritis is very likely to be related with these ESBL producing pathogenic *E. coli*. In addition, these isolates had similar antibiotic resistance and virulence factor profiles compared with human clinical isolates, showing that they may transmit between animals and humans to cause infectious diseases.

## Author Contributions

ZM, KG, and KJ designed the study. ZM, MK, and KG collected the samples. ZM, AG, and KJ analyzed the data. ZM and KJ drafted the manuscript. ZM, AG, KG, and KJ finalized this manuscript.

## Conflict of Interest Statement

The authors declare that the research was conducted in the absence of any commercial or financial relationships that could be construed as a potential conflict of interest.
